# Diagnosis and Treatment in a Tertiary Hospital of a Series of Complex Genital Malformations Corresponding to Double Uterus with Obstructed Hemivagina and Ipsilateral Renal Agenesis

**DOI:** 10.1155/2018/3806856

**Published:** 2018-12-02

**Authors:** Júlia Kefalás Troncon, Júlio César Rosa-e-Silva, Rayssa Miranda, Francisco José Candido-dos-Reis, Omero Benedicto Poli-Neto, Antônio Alberto Nogueira

**Affiliations:** ^1^MSc in Obstetrics and Gynecology, Department of Obstetrics and Gynecology, Ribeirão Preto Medical School, University of São Paulo, Ribeirão Preto, SP, Brazil; ^2^Associate Professor, Department of Obstetrics and Gynecology, Ribeirão Preto Medical School, University of São Paulo, Ribeirão Preto (SP), Brazil; ^3^Undergraduate student, Ribeirão Preto Medical School, University of São Paulo, Ribeirão Preto, SP, Brazil; ^4^Associate Professor, Department of Obstetrics and Gynecology, Ribeirão Preto Medical School, University of São Paulo, Ribeirão Preto, SP, Brazil; ^5^PhD, Professor, Department of Obstetrics and Gynecology, Ribeirão Preto Medical School, University of São Paulo, Ribeirão Preto, SP, Brazil

## Abstract

**Aim:**

To evaluate the clinical features, diagnostic routine, treatment, and prognosis of patients with double uterus with obstructed hemivagina and ipsilateral renal agenesis at a University Hospital.

**Methods:**

A retrospective study analyzing the medical charts of outpatients with similar complex genital malformations seen at the University Hospital of the Ribeirão Preto Medical School from 1994 to 2015.

**Results:**

Fourteen patients were included in this retrospective study, all presenting with double uterus with obstructed hemivagina and ipsilateral renal agenesis. The main symptom was dysmenorrhea occurring shortly after menarche, and pelvic ultrasound was the examination of choice. The treatment consisted of resection of the vaginal septum, complemented by an abdominal approach in 5 cases. Complications of the syndrome observed in this case series included severe endometriosis, pelvic abscess, need for hysterectomy, and salpingectomy.

**Conclusions:**

Severe dysmenorrhea shortly after menarche is a typical symptom of this kind of malformation, even though the diagnosis of patients who present with fistulization of the vaginal septum can be delayed due to milder clinical features. Pelvic ultrasound can be considered the first-choice examination in diagnostic routine. Relief of pain and prevention of complications can be achieved successfully in most cases by resection of the vaginal septum.

## 1. Introduction

The presence of double uterus with obstructed hemivagina and ipsilateral renal agenesis, also referred to as Herlyn-Werner-Wünderlich (HWW) syndrome, or OHVIRA syndrome, is a type of complex anomaly associated with changes in the fusion of the müllerian ducts, with different degrees of uterine duplicity (class III of Buttran and Gibbons) [1, 2], in addition to obstruction of the hemivagina and ipsilateral renal agenesis [3], corresponding to a concomitant Wolffian abnormality. This malformation more frequently courses with a uterus didelphys with partial vaginal duplication forming a transverse septum and obstruction of menstrual flow in this hemivagina associated with the ipsilateral renal agenesis. However, it may also be associated with a bicornuate bicollis uterus or a septate uterus with septation extending to the cervix and the vagina or also with unilateral cervical atresia [4]. Whatever the type of malformation, in all cases there is obstruction of the unilateral hemivagina and ipsilateral renal agenesis, so that the condition is also known as obstructed hemivagina and ipsilateral renal agenesis (OHVIRA).

Müllerian malformations are frequently associated with urinary malformations due to the intimate relationship between the müllerian ducts and the Wolff ducts during the embryonic period. If the Wolff duct fails to develop on one side, with the consequent occurrence of renal agenesis, the ipsilateral müllerian duct will develop in a more lateralized manner, thus impairing its fusion with the contralateral müllerian duct. In addition, the malformed hemivagina will not join the urogenital sinus in an appropriate manner, resulting in obstruction [3, 4]. Therefore, apparently this malformation results from anomalies of both Müllerian and Wolffian (or mesonephric) origin. That is, renal agenesis and disruption of vaginal development occur if the mesonephric ducts do not play their proper role. Concomitantly, the induction of the Müllerian by the mesonephric ducts is also disrupted, resulting in nonabsorption defects as the uterine duplicate [5, 6].

The real prevalence of this anomaly is difficult to measure since in general the diagnosis is not made during a clinical gynecological examination but rather during some surgical procedure or by imaging evaluation. The prevalence is higher among groups with a specific condition such as infertility or endometriosis than among unselected populations. However, the estimated incidence is 0.1 to 0.8% and the condition is usually discovered after menarche by causing progressive dysmenorrhea due to the presence of the transverse vaginal septum which prevents blood flow through the vaginal canal, causing the formation of hematocolpos [7–9]. In view of the accumulation of menstrual blood that is not discharged due to the septum, at diagnosis the condition may also appear as a pelvic mass. In addition, due to the obstruction of menstrual flow that potentially intensifies tubal reflux, the patient may develop the more severe forms of endometriosis. In cases of hematocolpos fistulization through the vaginal septum, the complaint may be fetid leukorrhea [3, 10]. Asymptomatic or barely symptomatic cases are associated with the form of anomaly that involves communication between the two uterine horns or an incomplete transverse septum. The condition is also associated with signs and symptoms of infertility, although pregnancy can occur in the normal uterine horn and in the previously obstructed horn after surgical intervention.

The objective of the present study was to analyze the clinical signs and symptoms, the procedures used at diagnosis and the short- and long-term prognosis of patients with this type of malformation seen in our service that could be compatible with the also called Herlyn-Werner-Wünderlich (HWWS) or OHVIRA Syndrome.

## 2. Methods

This was a retrospective study based on the survey of the medical records of patients seen from 1994 to 2015 at the Human Reproduction, Gynecological Endoscopy and Children's and Pubertal Outpatient Clinics of the Department of Gynecology and Obstetrics of FMRP-USP with a diagnosis of double uterus with obstructed hemivagina and ipsilateral renal agenesis, according to diagnostic criteria established in the scientific literature [1–4].

Date and age at first visit, symptoms, clinical examination, subsidiary exams, treatment instituted, associated pregnancies, and their results were obtained from the medical records.

## 3. Results

The data of 14 patients seen at the service during the follow-up period and fulfilling the diagnostic criteria of this kind of malformation were obtained from the medical records. Data regarding age and clinical signs and symptoms at the time of diagnosis, type of anatomical malformation, treatment, and the occurrence or absence of pregnancy during follow-up are presented in Table 1.

Most of the patients analyzed sought gynecological care a short time after menarche. Most patients had dysmenorrhea as the main clinical manifestation. In the initial approach, ultrasound was the examination of choice and was performed in almost all cases.

Regarding treatment, in addition to exeresis of the vaginal septum, 6 patients required a joint abdominal approach. Three patients underwent salpingectomy due to large tubal dilation associated with hematosalpinxs. One patient underwent hysterectomy three months after the septum exeresis due to infectious complications, one underwent salpingectomy and oophorectomy due to tube ovarian abscess, and one patient was submitted to salpingectomy due to pyosalpinx associated with rudimentary horn exeresis.

We observed not infrequent complications associated with this entity, among them 2 cases of severe endometriosis, 2 cases of abscess, 1 case of hysterectomy, and 4 cases requiring salpingectomy. In these cases the complications were not associated with age, the duration of symptoms, or type of surgical approach. Only 3 of the 14 patients studied reported pregnancy during a ten-year follow-up. But it is important to emphasize that conservative treatment with exclusive vaginal septum exeresis is the preferred treatment.

## 4. Discussion

The main symptom detected among the patients was dysmenorrhea and pelvic pain due to the presence of an obstructed hemivagina that resulted in the accumulation of metabolized blood, causing hematocolpos, hematometra, and hematosalpinx. Dysmenorrhea was reported to be moderate to intense and the patients with a less symptomatic condition had communication between the uterine horns or an incomplete or fistulized vaginal septum. In one of the most extensive reviews of published cases, 94% of the patients complained about dysmenorrhea, 14% about vaginal discharge, and 2% about an acute abdomen [4].

The gynecological examination and investigation confirmed the diagnosis of uterus didelphys, transverse vaginal septum, and ipsilateral renal agenesis with hematocolpos, representing the most common form of the so-called Herlyn-Werner-Wünderlich syndrome. The exam most frequently used and most efficient for the diagnosis of the reproductive apparatus was ultrasound, applied to 13 of the 14 patients. The only exception was a patient who exhibited an infectious complication with purulent secretion through the vagina. In the present series, contrast radiography was used to confirm renal agenesis. Diagnostic hysteroscopy with vaginoscopy and diagnostic laparoscopy were used in the more complex cases. Magnetic resonance was carried out in 4 patients but did not modify the diagnosis or the management in this case series. Ultrasound is the first line examination for the complementary evaluation of these patients, with the primary condition being dysmenorrhea. The magnetic resonance can be considered as second-line examination because of the cost-effectiveness; however in cases of complex müllerian malformations it can be extremely helpful [11].

The literature has extensively reported and confirmed the use of imaging methods such as ultrasound and magnetic resonance for the diagnosis of both müllerian and urinary tract malformations [3, 10], and laparoscopy continues to play an important role on the diagnosis of pelvic diseases and on the identification of complications such as endometriosis or pelvic abscesses.

Complicated evolution of the cases was due to either infection or endometriosis. Both occur in consequence of the existing obstruction to the menstrual flow: in the first case, when this accumulation of menstrual blood becomes infected and in the case of endometriosis due to menstrual blood reflux into the abdominal cavity.

**Figure 1:**
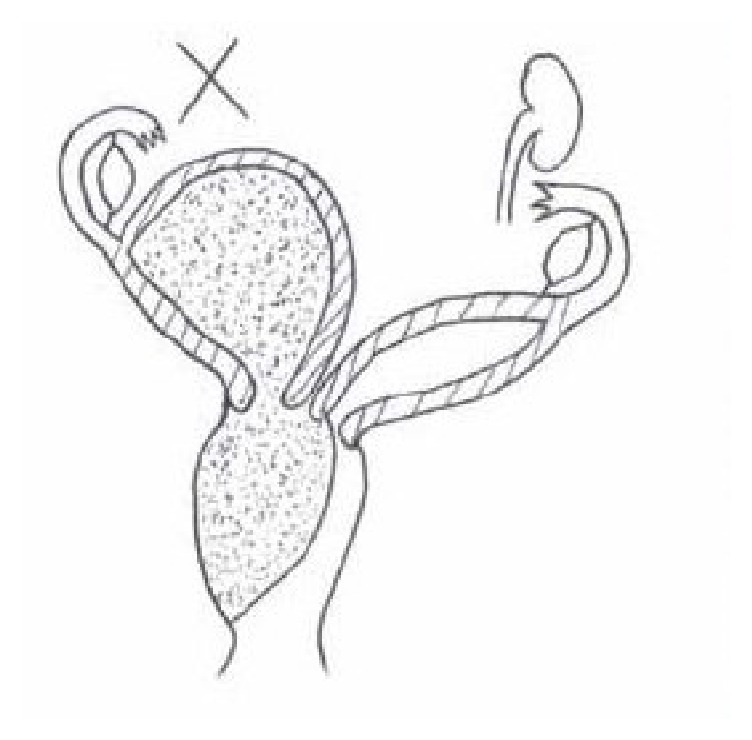
Genital malformation with uterus didelphys and right renal agenesis characteristic of the syndrome.

Patient treatment consisted of exeresis of the vaginal septum and drainage of hematocolpos, with consequent relief of symptoms. This approach is resolutive for cases of classical presentation as illustrated in Figure 1. Abdominal surgical intervention is usually unnecessary and, if indicated, is preferentially performed by the laparoscopic route. Other possible anatomical variations may require an individualized therapeutic approach. The primary objective of treatment, which involved relief of symptoms and the prevention of complications, was achieved. Resolution of symptoms occurred in most patients with the removal of the vaginal septum and three patients became pregnant without the need for additional treatment.

## 5. Conclusion

The main clinical manifestation observed in the patients was progressive dysmenorrhea after menarche. However, the diagnosis of patients with fistulization of the vaginal septum or communication between the uterine horns was delayed since their dysmenorrhea was less severe.

Although this is a rare condition, a high index of suspicion can lead to early diagnosis and timely intervention. It is also important to remember that, whenever malformation of müllerian ducts is diagnosed, it is essential to check for the associated renal malformations once they often coexist.

## Figures and Tables

**Table 1:** Description of the 14 cases of diagnosed HWWS.

**Case**	**Age at 1st visit**	**Clinical signs and symptoms**	**Diagnosis**	**Treatment**	**Pregnancy**
1	11	Progressively worsening dysmenorrhea persisting after the end of the menstrual flow	Uterus didelphys, complete transverse vaginal septum, R renal agenesis, R hematometra	Exeresis of the vaginal septum with discharge of chocolate brown blood	G1P1A0 (preterm pregnancy at 17 years)

2	13	Irregular cycles accompanied by dysmenorrhea since menarche	Uterus didelphys, complete transverse vaginal septum, R renal agenesis, R hematocolpos	Exeresis of the vaginal septum and drainage of hematocolpos	G0

3	24	Purulent and constant vaginal secretion	Uterus didelphys, transverse vaginal septum, R renal agenesis, purulent secretion	Resection of the transverse vaginal septum on the right and abscess drainage	G0

4	14	Irregular cycles with severe dysmenorrhea and difficulty urinating	Uterus didelphys, transverse vaginal septum, L renal agenesis, L hematometra	Resection of the vaginal septum, drainage of hematometra and L salpingectomy	G0

5	23	Daily bleeding associated with chronic pelvic pain of moderate intensity and dysmenorrhea	Uterus didelphys, transverse vaginal septum, L renal agenesis hematometra	Resection of the vaginal septum, drainage of hematometra and L salpingectomy	G0

6	13	Progressive dysmenorrhea since menarche	Uterus didelphys, transverse vaginal septum, R renal agenesis, hematocolpos (dilated cervix, body and right uterine tube)	Opening of the vaginal septum, drainage of hematocolpos and total abdominal hysterectomy 3 months after septum exeresis	G0

7	11	Progressively worsening dysmenorrhea persisting after the end of the menstrual flow	Uterus didelphys, transverse vaginal septum, L renal agenesis, hematocolpos, hematosalpinx	Exeresis of the vaginal septum, drainage of hematocolpos and salpingectomy	G0

8	12	Continuous pain in the hypogastrium arising with the menstrual flow	Uterus didelphys, transverse vaginal septum, L renal agenesis, hematocolpos	Exeresis of the vaginal septum, drainage of hematocolpos, oophorectomy and salpingectomy	G0

9	13	Progressive dysmenorrhea	Uterus didelphys, transverse vaginal septum, R renal agenesis, hydrosalpinx	Exeresis of R rudimentary horn and pyosalpinx	G0

10	13	Extensive vaginal bleeding and pelvic pain in inguinal regions	Uterus didelphys, transverse vaginal septum, R renal and ureteral agenesis	Exeresis of the vaginal septum	G0

11	29	Constant and progressive pain in a hypogastric region during the perimenstrual period	Uterus didelphys, transverse vaginal septum, R renal agenesis, hematocolpos, pelvic endometriosis	Exeresis of the vaginal septum and drainage of hematocolpos	G1P1A0 (cesarean delivery of a preterm liveborn at 35 weeks)

12	13	Increased menstrual flow since menarche	Uterus didelphys, transverse vaginal septum, L renal agenesis, presence of purulent content	Exeresis of the vaginal septum	G0

13	16	Vaginal discharge, blood clots after menstruation and intense menstrual cramps	Uterus didelphys, transverse vaginal septum, R renal agenesis, R hydrosalpinx	Exeresis of the vaginal septum	G4P3A1

14	21	Progressive dysmenorrhea and severe pelvic endometriosis	Uterus didelphys, transverse vaginal septum, R renal agenesis	Exeresis of the vaginal septum	G0

## Data Availability

The data used to support the findings of this study are available from the corresponding author upon request.
